# Expression and genomic organization of zonadhesin-like genes in three species of fish give insight into the evolutionary history of a mosaic protein

**DOI:** 10.1186/1471-2164-6-165

**Published:** 2005-11-22

**Authors:** Peter ND Hunt, Michael D Wilson, Kristian R von Schalburg, William S Davidson, Ben F Koop

**Affiliations:** 1 Centre for Biomedical Research, University of Victoria, Victoria, British Columbia V8W 3N5, Canada; 2 Department of Molecular Biology and Biochemistry, Simon Fraser University, Burnaby, British Columbia V5A 1S6, Canada

## Abstract

**Background:**

The mosaic sperm protein zonadhesin (ZAN) has been characterized in mammals and is implicated in species-specific egg-sperm binding interactions. The genomic structure and testes-specific expression of zonadhesin is known for many mammalian species. All zonadhesin genes characterized to date consist of meprin A5 antigen receptor tyrosine phosphatase mu (MAM) domains, mucin tandem repeats, and von Willebrand (VWD) adhesion domains. Here we investigate the genomic structure and expression of zonadhesin-like genes in three species of fish.

**Results:**

The cDNA and corresponding genomic locus of a zonadhesin-like gene (*zlg*) in Atlantic salmon (*Salmo salar*) were sequenced. Zlg is similar in adhesion domain content to mammalian zonadhesin; however, the domain order is altered. Analysis of puffer fish (*Takifugu rubripes*) and zebrafish (*Danio rerio*) sequence data identified zonadhesin (*zan*) genes that share the same domain order, content, and a conserved syntenic relationship with mammalian zonadhesin. A zonadhesin-like gene in *D. rerio *was also identified. Unlike mammalian zonadhesin, *D. rerio zan *and *S. salar zlg *were expressed in the gut and not in the testes.

**Conclusion:**

We characterized likely orthologs of zonadhesin in both *T. rubripes *and *D. rerio *and uncovered zonadhesin-like genes in *S. salar *and *D. rerio*. Each of these genes contains MAM, mucin, and VWD domains. While these domains are associated with several proteins that show prominent gut expression, their combination is unique to zonadhesin and zonadhesin-like genes in vertebrates. The expression patterns of fish zonadhesin and zonadhesin-like genes suggest that the reproductive role of zonadhesin evolved later in the mammalian lineage.

## Background

Molecules that are directly involved in reproduction are often subject to rapid evolutionary change [1]. Zonadhesin (ZAN) is one such molecule that has undergone domain expansion [2,3] and positive selection [4,5] in mammals. ZAN is a multi-domain sperm protein that is implicated in the species-specific binding of egg and sperm. Porcine (*Sus scrofa*) ZAN was first described by Hardy *et al*. [6] as a protein expressed by developing sperm that would bind to the zona pellucida of the egg. Since its initial discovery, zonadhesin has been identified in several other mammals, including mouse, human [2] and rabbit [7]. Recent data suggest the processed zonadhesin localizes to the acrosomal matrix and binds the zona pellucida during the acrosome reaction [8].

The discrete domains of mosaic proteins are known to be important in the evolution of new genes. Domain subunits can be rearranged, duplicated or deleted to produce a variety of proteins with different functions [9]. Zonadhesin structure is unique in its combination of protein domains. All mammalian zonadhesin genes are predicted to encode: a signal peptide, a multiple meprin A5 antigen receptor tyrosine phosphatase mu (MAM) domain, multiple trypsin-like inhibitor (TIL) domains, multiple von Willebrand D (VWD) cell adhesion domains, multiple hepta-peptide repeats that form the mucin domain, multiple epidermal growth factor (EGF) domains, a single transmembrane domain and short intracellular domain at the carboxyl terminus. The domain order is the same for all mammals studied, with the main difference being the number of MAM and VWD domains. These individual domains each have a particular function and are found in many other mosaic proteins.

The extracellular VWD domain occurs in a family of immediate-early genes that are growth regulators and is thought to have an adhesive function. This modular domain is found in a variety of mosaic proteins including von Willebrand factor [10], apolipoprotein B, vitellogenins, microsomal triglyceride transfer protein (MTP) [11], and mucins [12]. Biochemical studies of pig zonadhesin have shown that the ZAN precursor is processed and the MAM domains are removed leaving the VWD domain to interact with the zona pellucida [13]. While the role of the VWD in sperm-egg binding has been addressed, the role of the mucin-repeats and the MAM domains is still unknown.

The mucin or MUC domain is the primary functional domain of mucin proteins. Mucins are a diverse group of heavily glycosylated proteins that are the major component of mucus. Mucins function to lubricate surfaces and are the first line of defence against pathogens [14]. Most of the secreted mucins contain a domain with sequence similarity to VWD and a domain composed of a variable number of tandem repeats that code for serine-, threonine- and proline-rich repeat peptides that are potential glycosylation sites [15]. Two mucin-like genes that are similar to zonadhesin include alpha-tectorin, which is involved in non-syndromical autosomal dominant hearing impairment [16,17], and Fc fragment of IgG binding protein (FCGBP). By virtue of sequence identity, the closest relative to mammalian zonadhesin is FCGBP and this similarity is mostly seen in the TIL and VWD domains. *FCGBP *is expressed in the mucosa of the small and large intestines, epithelial colon cells and the placenta and is thought to play a role in the mucosal immune system through the promotion of multivalent IgG and the trapping of antigen IgG complexes in the mucosa [18-20].

The 170 amino acid MAM domain distinguishes zonadhesin from other VWD and mucin repeat containing genes such as *FCGBP*. MAM domains have adhesive function and are found in several proteins including protein-tyrosine phosphatases, neuropilins and meprins. Meprins are metalloendopeptidases that have been found in the intestinal brush border and renal membranes of mammals [21]. While their role in the zonadhesin protein is unknown, MAM domains are important for multimerization [22] and have conserved cysteine residues that are responsible for covalent interactions [23].

When this study began, zonadhesin expression had only been described in the testes. Only zonadhesin was thought to encode MAM domains, mucin repeats, and VWD domains and no non-mammalian zonadhesin orthologs had been reported. For these reasons we were interested in Atlantic salmon (*Salmo salar*) zonadhesin-like ESTs from a gut-derived library that encoded the MAM, mucin and VWD domains. Here we describe the cDNA, genomic structure, and expression patterns of this Atlantic salmon zonadhesin-like gene. We also use comparative genomic and expression analyses to uncover additional zonadhesin-like genes, as well as orthologs of zonadhesin, in zebrafish (*Danio rerio*) and puffer fish (*Takifugu rubripes*).

## Results and discussion

### Characterization of a zonadhesin-like gene in *S. salar*

**Figure 1:**
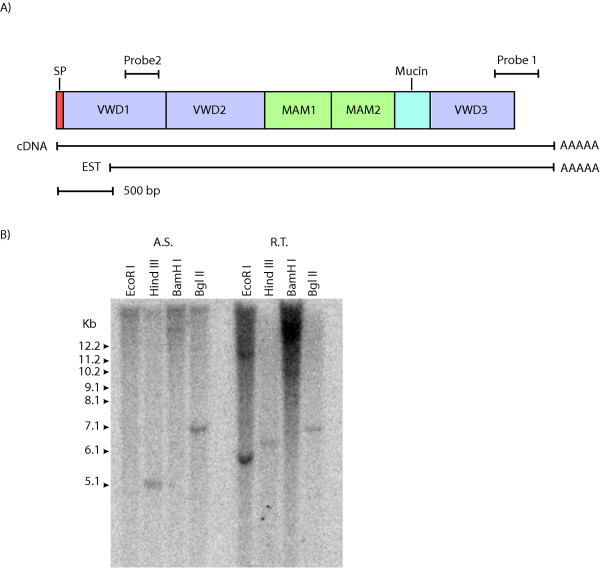
**Organization and Southern blot analysis of the Atlantic salmon zonadhesin-like gene (*zlg*)**. A) Domain structure, probe locations, and EST coverage of the Atlantic salmon Zlg. The three VWD domains, the two MAM domains and the mucin domain are shown. The signal peptide (SP) and poly (A) tail are also indicated. Probe 1 included 177 nucleotides of the 3'-UTR and 206 nucleotides of VWD3 in the coding region. Probe 2 included 233 nucleotides of the 3'-end of VWD1. The 4388 bp EST covers the full length of the cDNA sequence except for the 5'-UTR which is estimated to be approximately 200 bp by comparison to Northern blot data. B) Southern blot analysis of Atlantic salmon and rainbow trout genomic DNA. Gene copy number was assessed by genomic hybridization. Twenty micrograms of Atlantic salmon (A.S.) and rainbow trout (R.T.) genomic DNA were digested with four enzymes, *Eco*R I, *Hind *III, *Bam*H I and *Bgl *II and hybridized with radiolabeled probe 1 representing the 3'-end of the *zlg *mRNA.

A gene similar to mammalian zonadhesin was identified during an expressed sequence tag (EST) analysis of Atlantic salmon. We assembled this zonadhesin-like gene (*zlg*) from five overlapping ESTs [24]; the largest of which [GenBank: CK990464] was sequenced by primer walking from both directions and assembled to give a 4388 bp sequence. PCR primers were designed to the 3'-end of the EST and used to amplify a probe for hybridization to Atlantic salmon genomic DNA and bacterial artificial chromosome (BAC) library filters (Probe 1, Figure 1A).

Probing of 91,776 BAC clones on five Atlantic salmon genome BAC library filters [25] resulted in one positive BAC. This BAC (722P12) was subcloned, sequenced, and assembled into a 138,345 base pair contiguous sequence [GenBank: AY785950]. The assembly had more than 3000 high quality sequence reads and 10 fold sequence coverage in most regions. One gap of 500 bp was filled by PCR followed by sequencing from both directions. An *in silico *restriction digest matched experimental digests. The zonadhesin-like gene was the only gene found on this BAC. All other open reading frames were associated with LINE, SINE and transposon related repetitive elements.

Comparison of 722P12 against itself using Dotter and PipMaker [26] did not reveal any recent domain expansions or duplications. The Simple Modular Architecture Research Tool (SMART) [27] was used to identify conserved domains of the predicted protein (Figure 1A). The SMART algorithm was used to detect three VWD domains (amino acid positions 31–198, 415–578 and 1277–1498), a VWC domain at (position 365–425), and two MAM domains (positions 708–870 and 895–1056). This tool also located three low complexity regions that corresponded to the mucin domains predicted by the computer program NetOGlyc 3.1 [28]. These mucin domains occurred at positions 1091–1099 and 1115–1158 with a smaller low complexity region located between nucleotides 693 and 704. These results were corroborated using the InterPro domain prediction computer program [29]. The SMART and InterPro domain prediction tools, in agreement with Kyte-Doolittle hydropathy data, did not find any transmembrane domains in the salmon Zlg. In contrast, the SMART tool detected a transmembrane domain at the expected location for zonadhesins from other species.

Using two distinct probes (Figure 1A), only one copy of *zlg *was found in Atlantic salmon by Southern blot analysis (Figure 1B). However, two bands occurred when the same probes were used with rainbow trout genomic DNA (Figure 1B). Probe 1, which contained sequence homologous to the 3'-UTR of *zlg *(Figure 1), would be expected to be specific for this gene; however, probe 2 spanned VWD1 and could be expected to hybridize with related zonadhesin-like genes. Both probes gave similar results and only probe 1 data is shown in Figure 1B. Southern blot analysis did not reveal any other genes in Atlantic salmon other than *zlg*. This suggests that if another zonadhesin-like gene does exist in Atlantic salmon it is likely quite divergent from *zlg*.

**Figure 2:**
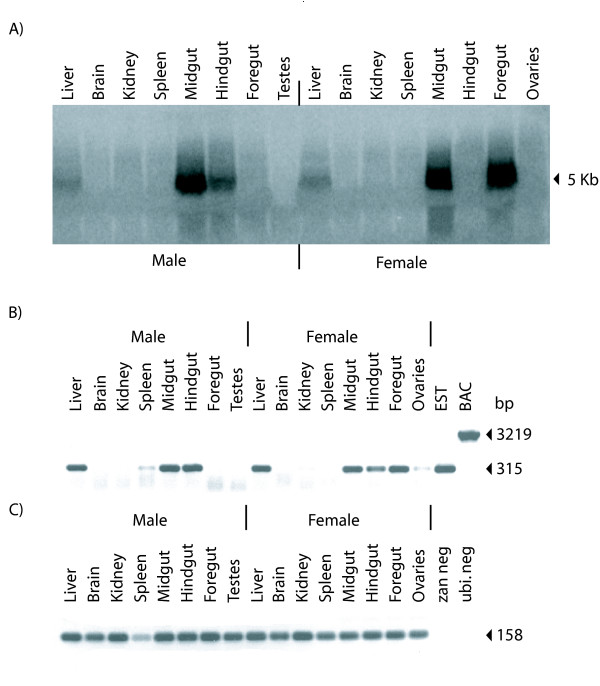
**Expression of the Atlantic salmon *zlg *mRNA**. A) Expression of the zonadhesin-like gene was analyzed in a variety of tissues. Ten micrograms of total RNA from liver, brain, spleen, kidney, midgut, hindgut, foregut and gonads from male and female Atlantic salmon was blotted on a positively charged nylon membrane and hybridized with radiolabeled probe 1 representing the 3'-end of the Atlantic salmon *zlg *mRNA. B) Analysis of the Atlantic salmon *zlg *tissue expression pattern by semi-quantitative reverse transcription PCR. *Zlg *primers ('probe 1' primer set) were used to test for the presence of the *zlg *cDNA. The EST to which the primers were designed [GenBank: CK990464] and BAC 722P12 were used as positive controls. The genomic region encompassed by the primers contains two introns of 395 and 441 bp yielding a 1219 bp band upon amplification. C) Ubiquitin primers were used as a positive control for mRNA presence. *zlg *(ZAN neg) and ubiquitin (Ubi neg) template-free negative controls were included.

The 5'-end of the *zlg *cDNA coding sequence was obtained by PCR using primers designed to the predicted translation start site on the BAC sequence. The final assembly of the cDNA was 4,791 bp [GenBank: AY785949]. The total length of the mRNA found by Northern blot was just over 5 Kb (Figure 2A). The additional length of the mRNA is likely comprised of the 5'-UTR of *zlg*. The cDNA aligned completely to the BAC and the 23 consecutive exons of the zonadhesin-like gene utilized canonical splice sites. The cDNA has a predicted ORF of 4,518 bases that encodes a 1,506 amino acid protein. The predicted protein starts with a methionine and has a putative signal peptide of 18 amino acids. A poly (A) signal of AATAAA was identified by Genscan [30,31] at 4770 bp from the start codon of the cDNA, 241 bp downstream of the stop codon.

### Salmo salar *zlg *expression

Semi-quantitative reverse transcription PCR was used to identify tissues expressing the *zlg *mRNA (Figure 2B). Liver, brain, kidney, spleen, foregut, midgut, hindgut and gonads were taken from one male and one female Atlantic salmon. Male and female salmon showed expression in the liver, midgut and hindgut. However, expression in the spleen only occurred in the male and expression in the foregut only occurred in the female. A weak band was present in the ovarian sample and expression was not observed in the testes. This gene does not appear to be expressed in either male or female brain or kidney.

Northern blot results were similar to the RT-PCR results and showed a single band in male midgut and hindgut, and female midgut and foregut (Figure 2A). A weak band was seen in the Northern blot analysis of liver from both male and female fish (Figure 2A). Unlike the RT-PCR results, Northern blot analysis did not detect a transcript in the male spleen and female hindgut. This discrepancy could be due to the higher sensitivity of the PCR experiment. The *zlg *expression pattern differed from the testes-specific expression known for mammalian zonadhesin. To clarify the relationship between *zan *and *zan*-like genes, we looked for related genes in the genomes of other fish.

### Genomic analysis of the zebrafish *zan *locus

**Figure 3:**
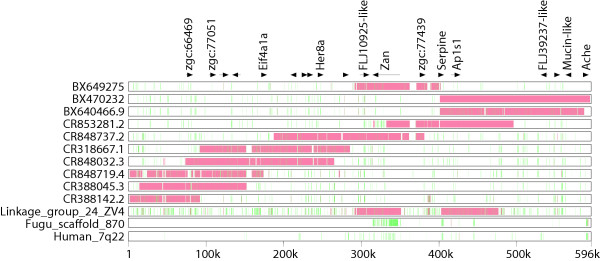
**Alignment of zebrafish BACs to the linkage group 7 of the ZV4 whole genome shotgun assembly**. Approximately 600 Kb of the zonadhesin locus from the ZV4 assembly is shown on the x-axis. Alignments of this region to zebrafish BACs, the puffer fish (fugu) *zan *locus (Scaffold 860) and human 7q22 were performed using MultiPipMaker. Regions of high conservation consisting of gap-free alignments of at least 100 bp and 70 percent nucleotide sequence identity are shown in red and other locally aligned regions are shown in green. RepeatMasker [45] was used to mask zebrafish repeats before the alignments were made.

An initial inspection of the ZV4 (September 6^th^, 2004 release) whole genome shotgun (WGS) assembly of the *D. rerio *genome suggested there are two copies of zonadhesin that exist at two distinct loci. One copy is found at linkage group 7 (scaffold 588) and consists entirely of whole genome shotgun reads. The second copy is found at linkage group 24 (scaffold 1965) and resides within a completely sequenced BAC from the CHORI-211 library [GenBank: BX649275]. The ZV3 assembly contained only one copy of zonadhesin that also assembles into linkage group 7. It is important to note that the ZV3 assembly consisted entirely of whole genome shotgun reads; the ZV4 assembly incorporated finished BAC sequence into the ZV3 assembly; and that both assemblies are considered 'pre-assemblies' that need to be analyzed with caution. The location of zonadhesin at linkage group 7 is supported by the fact that other genes with significant similarity to human chromosome 7q22 map to the same region of the *D*.*rerio *genome. These genes include acetylcholine esterase, serpine, *AP1S1*, unnamed product FLJ39237, unnamed protein product FLJ10925 and mucin (Figure 3).

The regions surrounding the *zan *locus at both linkage groups were inspected for possible segmental duplications. BAC clones (both finished and unfinished) that aligned to a 600 kb region surrounding the *zan *locus at linkage group 7, or a 1 MB region at linkage group 24, were obtained and aligned to both loci using the BLASTZ algorithm through the MultiPipMaker web server. Our analysis showed that the zonadhesin-containing BAC BX649275 integrated completely into linkage group 7 and was ≅ 97% identical overall, not including indels. Seven additional BACs from the DKey library aligned to the scaffold and produced an acceptable tile through the entire *zan *locus (Figure 3). In contrast, only a portion of BAC BX649275 and a portion of BX640466.9 aligned to the *zan *locus at linkage group 24. This is suggestive of an assembly artifact that resulted in the assignment of a second zonadhesin gene to linkage group 24.

The differences between individual BACs, and between BACs and linkage group 7, can be as high as 3%. This amount of polymorphism is higher than the 0.5% polymorphism rate expected from the whole genome shotgun sequence, which came from approximately 1000, 5 day old embryos [32]. Despite this high rate of polymorphism, the existence of one zonadhesin locus at linkage group 7 is supported by the large tile of overlapping clones at linkage group 7, and the rapidly evolving nature of zonadhesin genes [1].

### Prediction of the zebrafish *zan *transcript and domain structure

**Figure 4:**
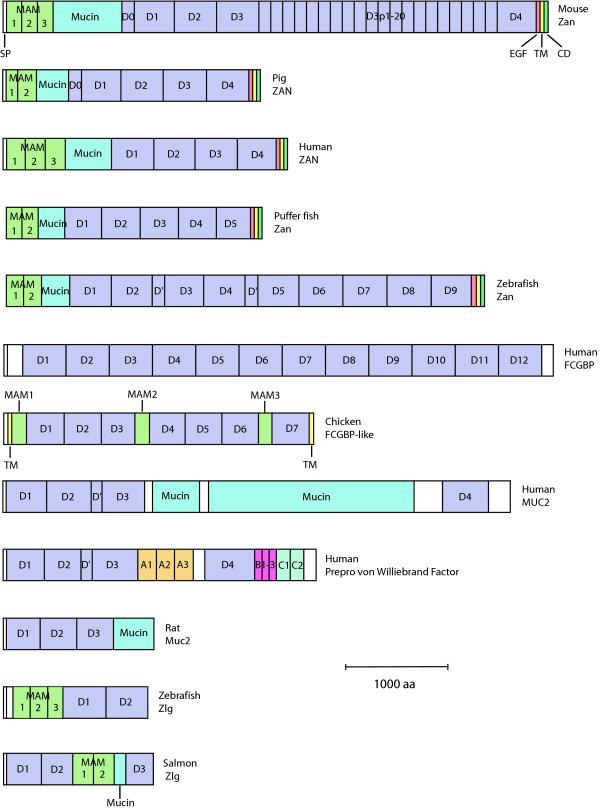
**Domain structures of representative zonadhesins and related proteins**. Mouse [GenBank: NP035871.1], pig [GenBank: Q28983], human [GenBank: Q9Y493], puffer fish and zebrafish zonadhesins are shown with the salmon and zebrafish Zlg. The human MUC2 [GenBank: L21998.1], human prepro von Willebrand factor [GenBank: P04275] and rat (*Rattus norvegicus*) MUC2 [GenBank: Q62635] and human and chicken FCGBP proteins [GenBank: NP003881.1 and XP422715.1] are also included. Signal peptide sequences (SP) are drawn as a white box and are found at the N-terminus of all proteins except for puffer fish and zebrafish Zan. All the zonadhesins have an epidermal growth factor (EGF) domain (in red), a transmembrane (TM) domain (in yellow) and a cytoplasmic domain (CD) (in green) which are labeled on the mouse Zan protein. The MAM and mucin domains are indicated and the VWD domains are represented by 'D'. The partial D3 domains of the mouse are represented by D3p1-20. Other partial VWD domains are denoted D0 or D' as found in the current literature. It is important to note that although this domain representation is consistent with previous representations of zonadhesin and other VWD containing proteins, other domain types such as von Willebrand C (VWC) and trypsin inhibitor-like (TILa) domains have not been included here.

We further analysed the zonadhesin gene found on the completely sequenced BAC (BX649275). The 4,616 amino acid translation product of the putative *zan *gene lacked a signal peptide, but contained the domain structure of: two MAM domains; a mucin domain; nine VWD domains; and a transmembrane region. This domain organization is typical of zonadhesin (Figure 4). The entire zonadhesin gene was also contained within 13 unordered pieces from the clone DKey-3K24 [GenBank: CR848737.2]. From this clone we obtained a third putative *zan *transcript that was most similar to the complete *zan *sequence from BAC BX649275. The predicted cDNA from CR848737.2 differs from the cDNA of BX649275 by 0.8%. The putative ZAN protein encoded by BAC CR848737.2 has a domain structure the same as the one encoded by the putative *zan *transcript of BAC BX649275.

Zebrafish zonadhesin has expanded its VWD domains through recent tandem duplication. The VWD domains 5, 6 and a fragment of 7 are encoded in 17 exons (45–61). These exons have identical sizes to the exons (11–27 and 28–44) encoding the first four VWD domains. Furthermore, each group of 17 exons are symmetrical and flanked by phase '1' introns, which is evidence for recent domain expansion. Pair-wise alignment of BAC BX649275 against itself revealed these exons are found in three ≈ 5 kb blocks that are 85–87% identical. The *zan *locus at linkage group 7 only contains two of these ≈ 5 kb repeats. It is possible the third repeat was collapsed in the whole genome shotgun assembly process or the presence of two repetitive blocks is a true population variant.

### *Danio rerio *zonadhesin expression

**Figure 5:**
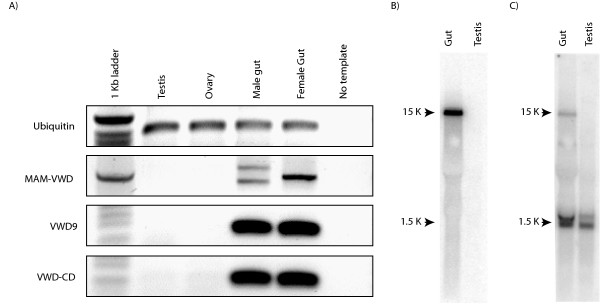
**Zebrafish zonadhesin mRNA expression**. A) Semi-quantitative reverse transcription PCR analysis of zebrafish tissues. Three primer sets were designed against zonadhesin specific sites. The amplicons of the first primer set crossed the mucin domain stretching from the MAM domains to the first VWD domain (MAM-VWD). The second primer set amplified within the VWD9 domain (VWD9). Products of the third primer set stretched from the last VWD domain to the cytoplasmic domain (VWD9-CD). Ubiquitin primers were used as a positive control and a template-free reaction was included as a negative control. B-C) Expression of zebrafish zonadhesin analyzed by Northern blot. B) Expression of zonadhesin was investigated in the zebrafish testis and gut. Five micrograms of total RNA from each tissue was blotted on a positively charged nylon membrane and hybridized with a DNA probe encompassing the zebrafish EGF to CD region of *zan*. C) The same Northern blot membrane was reprobed with zebrafish alpha-tubulin as a positive control.

To compare the expression pattern and verify the transcript size of the predicted zebrafish *zan *we extracted gut and testes RNA and performed RT-PCR and Northern blot analysis. Three different PCR primer sets were designed against three regions of the predicted *zan *cDNA. The first primer set was designed to amplify the exons encoding the MAM domains through to the first VWD domain. This primer set produced a doublet in both male and female (the larger band in the female was very faint) (Figure 5A). The female bands were approximately 50 bp larger than the male bands. The differences between individuals may be due to a variation in mucin domain length since mucin domains have been shown to be variable in other genes [12]. The second primer set flanked the ninth VWD domain and the third stretched from the epidermal growth factor domain to the cytoplasmic domain. Each primer set identified a zonadhesin transcript in the gut, but not in the testes (Figure 5A). These results were corroborated by Northern blot analysis that found a single transcript of ≅ 15 Kb in the gut but not in the testes (Figure 5B and 5C). This ≅ 15 kb transcript correlates to the mRNA length predicted from the genomic sequence and supports the existence of a single zonadhesin gene in zebrafish.

### Genomic prediction *T. rubripes zan *cDNA transcript and domain structure

Analysis of the puffer fish genome assembly release 2 (SCAFFOLDS 17 05 02; [33]) revealed a putative zonadhesin gene in the same contig (scaffold 870) as the *ache *gene. This syntenic relationship was also found at the mammalian and zebrafish *zan *loci (Figure 3). However, Scaffold 870 has been split in the current Fugu genome project release (MAYFFOLDS) leaving zonadhesin in a gap-free region of scaffold 2,670 without *ache*.

Puffer fish zonadhesin was predicted to contain 47 exons that coded for a protein of 2,525 amino acids. The predicted zonadhesin protein contained two MAM domains at the N-terminus, a mucin domain, five VWD domains, an EGF domain, a transmembrane domain and a short cytoplasmic domain (Figure 4). This protein has the same domains in the same order as human zonadhesin. No signal peptide was identified in the puffer fish zonadhesin. However, this sequence may be incomplete since it was found at the end of the scaffold sequence.

### Expression of *T. rubripes *zonadhesin

Evidence from the GenBank database suggests that puffer fish zonadhesin is also expressed in the gut. Sequences can be found that have been isolated from gut-specific libraries [GenBank: CA591505, CA588342 and CA588225], but there have been no zonadhesin sequences found with testes-specific expression.

### Structural similarity of *D. rerio *and *T. rubripes *to mammalian *zan *genes

Although fish and mammals have not shared a common ancestor for an estimated 450 million years [34], the domain structure of zonadhesin has been highly conserved (Figure 4). However, domain numbers between species are variable and this variability appears to have been influenced by tandem duplication. Tandem duplications have occurred in both mammalian and fish species and are most prevalent in the VWD domain region (Figure 4). It is this region which, at least in mammals, has been shown to be important for zona pellucida binding [35]. While multiple VWD domains are found in all characterized zonadhesins, recent expansion of this domain is seen in the mouse and in the zebrafish. Repeated tandem duplication in the mouse zonadhesin gene resulted in 20 copies of a two-exon segment encoding a partial VWD3 domain that increased the length of the protein by over 2000 amino acids [2,3]. In zebrafish, the double duplication of two domains homologous to the puffer fish VWD1 and VWD2, as well as a portion of the VWD3 domain (containing 17 VWD-coding exons), resulted in an additional 34 exons that encoded 4 additional full VWD domains and two partial VWD domains in the zebrafish Zan (Figure 4).

The ancestral zonadhesin likely looked similar to the puffer fish Zan as it is very similar in length and domain structure to most mammalian zonadhesins (Figure 4). The puffer fish gene also has slightly higher identity with the human zonadhesin gene at 52% (indels removed) compared with the 50% identity between zebrafish and human whereas the zebrafish and puffer fish genes are 62% identical.

**Figure 6:**
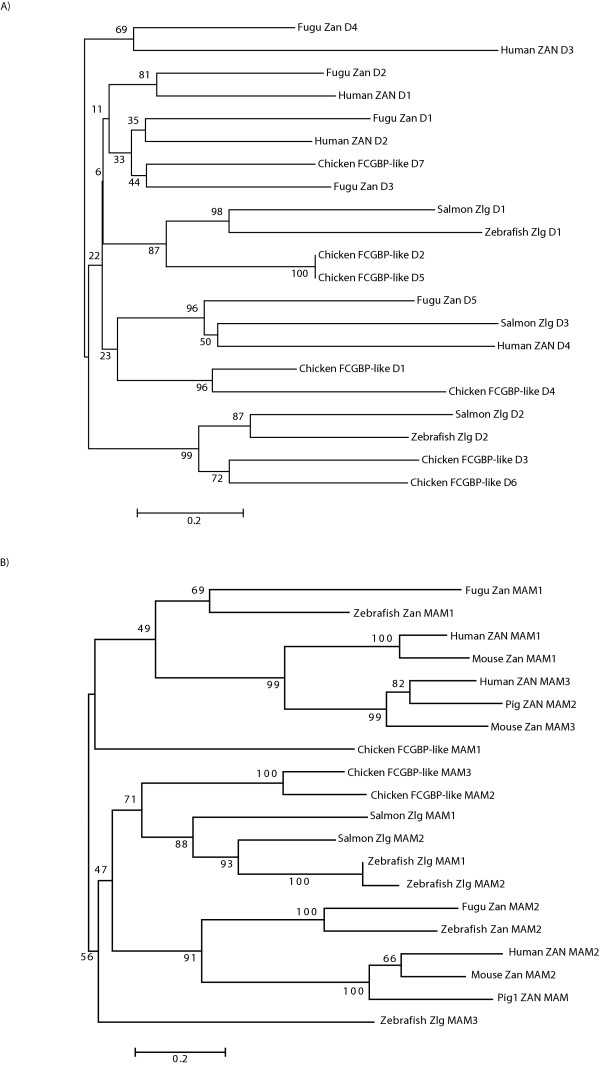
**Phylogeny of the VWD and MAM domains of zonadhesin and zonadhesin-like genes**. A) Human and puffer fish zonadhesin VWDs were aligned with the VWD domains of salmon and zebrafish Zlgs and chicken FCGBP-like protein. A neighbor-joining tree utilized 140 informative sites. B) Phylogeny of the MAM domains from puffer fish, zebrafish, pig, mouse and human ZANs, salmon and zebrafish Zlg and chicken FCGBP-like proteins are shown with a neighbor-joining tree. This phylogeny utilized 88 informative sites. Both phylogenies used gap-free alignments and a Poisson substitution correction model. Consensus trees based on 1000 pseudo-replicates are reported with the bootstrap support values indicated above the respective nodes. The scale is given in amino acid substitutions per site.

The five VWD domains of the puffer fish also cluster with the four in human (Figure 6A). The last four VWD domains of puffer fish Zan seem to be homologous to the last four human domains; although, the first VWD domain of puffer fish also has high similarity to the second domain in human. This may be the result of an ancient duplication of the first two VWD domains in puffer fish and subsequent loss of the new first domain. This inheritance is similar to that of the MAM domains.

Puffer fish zonadhesin has two MAM domains, a structure matching the rabbit and pig proteins [7]. Neighbor-joining tree analysis of individual domains reveals that the MAM1 domain of puffer fish is most similar to the MAM1 and MAM3 domains of human, while the MAM2 domain of puffer fish groups with MAM2 of human (Figure 6B). This pattern of similarity could be explained by a duplication of both MAM domains in the human lineage and subsequent loss of the fourth domain. This phylogeny, in combination with the conserved domain order and synteny between fish and mammal zonadhesin loci, supports the orthologous relationship of these genes.

### Genomic and phylogenetic analysis of genes with a domain content similar to zonadhesin

Until this study, zonadhesin was generally thought to be unique in its domain content as no other genes were reported to contain MAM, mucin, TIL and VWD domains. However, the characterization of the salmon *zlg *revealed all of these domains, but in a different order (Figure 4). The expression of *zlg *is similar to zebrafish zonadhesin which, in addition to domain content, established a possible evolutionary link between these genes. Examination of GenBank sequences also revealed that zonadhesin-like genes have been found in gut tissues of other species; however, the automated annotation is based on the similarity of the TIL and VWD domains of *FCGBP*. For example, there are three human colon ESTs [GenBank: AI984139, AI983786 and AI983612] that are annotated as similar to zonadhesin; however, these genes align perfectly to the *FCGBP *gene at chromosome 19q13.2. Similarly, the only mouse colon EST that is annotated as similar to zonadhesin aligns to the *Fcgbp *gene in the orthologous region at mouse chromosome 7.

We looked for zonadhesin-like genes in puffer fish, zebrafish and chicken (*Gallus gallus*) genome projects. This search uncovered several regions with VWD-containing proteins without any detectable MAM domains, some of these possibly related to FCGBP proteins. One interesting exception was a zonadhesin-like gene found on zebrafish chromosome 2. This putative zebrafish *zlg *encodes a 1,308 amino acid protein containing three MAM domains, the first of which is flanked by short (15 amino acid) mucin-like low complexity regions. The MAM domains are followed by two VWD domains. This gene structure is reminiscent of *S. salar *Zlg and shows that a zonadhesin ortholog and a *zan*-like gene exist together in the zebrafish genome (Figure 4).

The search for zonadhesin-like genes in chicken (*Gallus gallus*) did not reveal an obvious zonadhesin ortholog but rather a prediction of a FCGBP-like protein residing on chromosome 9 [GenBank: XP422715.1]. We analysed the corresponding region of the *G. gallus *genome and utilized additional EST evidence and *in silico *predictions to obtain a putative transcript that encodes a 4,770 amino acid gene product. This gene product contains three MAM domains in addition to VWD and mucin-type O-glycosylation sites after each of the MAM domains. Overall, this MAM-mucin-TIL-VWD series of domains is reminiscent of all zonadhesins and is evidence for a common evolutionary origin of zonadhesin, the zonadhesin-like genes and the Fc fragment of IgG binding protein(*FCGBP*).

We extracted the MAM and VWD domains of representative zonadhesin and zonadhesin-like genes and performed a phylogenetic analysis (see Figures 6A and 6B respectively). In addition to the clustering of fish and mammalian zonadhesin, both phylogenetic trees suggest an evolutionary relationship among the zonadhesin-like genes. In particular, the zebrafish Zlg MAM1 and MAM2 domains grouped with the salmon Zlg MAMs as well as two of the chicken FCGBP-like MAM domains (Figure 6B). The grouping of the MAM domains of these proteins indicates that the zonadhesin-like genes represent a novel gene family that is distinct from zonadhesin.

Although the phylogeny is more complex with many nodes not well supported, the evolutionary relationship between the fish Zlgs and the chicken FCGBP-like gene was also observed for the VWD domain (Figure 6A). For example, the VWD1 domains of the fish Zlgs clustered with the VWD2 and VWD5 domains of the chicken FCGBP-like gene. A second clade consisting of the VWD2 domains of salmon and zebrafish Zlg, and the VWD3 and VWD6 domain of the chicken FCGBP-like gene also formed. The clade containing puffer fish ZAN VWD5, human ZAN VWD4 and salmon Zlg VWD3 also suggests that zonadhesin is closely related to the zonadhesin-like genes. The human FCGBP VWD domains all formed a single clade except for VWD1 which grouped with the chicken FCGBP-like VWD1 and 4 (data not shown). Overall, the phylogenies of the VWD and MAM domains combined with the expression patterns of: fish zonadhesins, fish and chicken zonadhesin-like genes, *FCGBP*, mucin, and several MAM containing proteins, suggest that these mosaic genes share a common ancestor.

### Zonadhesin in the context of other sperm-egg interacting proteins

Many mammalian zona pellucida adhesion molecule candidates appear to have evolved from different physiological processes. Well known examples of sperm proteins with enzymatic function that have been 'hijacked' into playing non-enzymatic roles in sperm-egg interactions include: B4GALT1/GalTase (beta 1,4 galactosyltransferase), SPAM1/PH-20 (hyaluronidase), HK1/ZRK/p95 (hexokinase), and ARSA/SLIP1 (aryl-sulfatase-A; reviewed by [36]). Evidence for immune-system hijacking events comes from the discovery of several complement system proteins in human spermatozoa, seminal plasma and follicular fluid (reviewed by [37]). The partial activation of the complement system (without engaging the membrane attack complex) in acrosome-reacted spermatozoa suggests how components of a conserved immune system pathway could play a new role in sperm-egg recognition [38]. Although the function of the fish zonadhesin and zonadhesin-like genes is not known, their expression in the gut and absence of expression in the testes, combined with their homology to gut-expressed genes of the mucosal immune system (i.e. *FCGBP *and mucin), suggest that zonadhesin was also 'hijacked' by the mammalian reproductive system.

## Conclusion

We identified zonadhesin genes in zebrafish and puffer fish that are similar in domain order and content to all known mammalian orthologs. Unlike all mammalian zonadhesin genes studied to date, zebrafish *zan *was expressed in the gut but not in the testes. In addition to these orthologs, we characterized zonadhesin-like genes (*zlg*) in Atlantic salmon, zebrafish and chicken. While the Atlantic salmon *zlg *contained the same domains found in zonadhesin, the order of these domains was altered and the expression was found predominantly in the gut and not in the testes. Overall, this suggests that zonadhesin's reproductive role evolved later in the mammalian lineage.

## Methods

An Atlantic salmon CHORI-214 bacterial artificial chromosome (BAC) library was obtained from BACPAC Resources, Children's Hospital Oakland Research Institute (CHORI) [25]. Five BAC library filters (13A-17A) were hybridized with a probe designed from a zonadhesin-like EST [GenBank: CK990464]. These five filters contained 91,776 BAC clones in a pTARBAC2.1 vector with an average size of 190 Kb. Each filter was estimated to represent the salmon genome once. Filter hybridizations were conducted as described by CHORI [39]. The PCR product that was used as a probe was generated by PCR (Invitrogen) using the manufacturer's protocol and the following primer set: 5'-GTGCCCATTGTAGGAAGGAA-3' and 5'-GGGGTTGAGGATTCTGGAG-3'. The probe was gel purified and end-labeled with γ^32^P-ATP (Amersham). Probed BAC library filters were visualized using a Molecular Dynamics Storm PhosphorImaging system.

BAC DNA was isolated by an alkaline lysis procedure using Nucleobond columns (Clontech) using the manufacturer's protocol. The isolated 722P12 BAC DNA was nebulized and the DNA was blunt-ended. The blunt-ended repaired DNA was size fractioned by electrophoresis and the gel region corresponding to 1200–3000 bp was excised and gel purified (Qiagen). The fragments were blunt-end ligated into pUC19 plasmid cut with Hinc II (NEB) and transformed into electrocompetent DH5α *E. coli *cells using a Bio-Rad Gene Pulser system. Extracted recombinant plasmid templates were sequenced on an ABI 3700 DNA sequencer. Bases were called using PHRED [40,41]. The resulting 3000+ high quality sequence reads were assembled using PHRAP [42] and then viewed and edited using Consed [43]. One gap of about 500 bp in the assembly was filled by designing primers to the contig ends followed by amplification of this BAC region by PCR and subsequent cloning and sequencing this fragment. Restriction digests of the isolated BAC were compared to *in silico *digests for assembly confirmation. BAC 722P12 was deposited in GenBank under the accession number AY785949.

Dotter [44] and PipMaker [26] were used to compare the BAC sequence to itself and to identify duplicated and repeated regions. Identification of other repeat elements was done with RepeatMasker [45] using repeat library 4.01 from Repbase [46]. Low complexity regions that corresponded to the mucin domains were predicted by the computer program NetOGlyc 3.1 [28]. Genscan was used to predict novel genes and gene structures [30,31]. Translated and untranslated BLAST searches were performed using 722P12 BAC as the query.

The Atlantic salmon *zlg *cDNA was partially sequenced by first completing a series of primer walks from the 5'- and 3'-ends to complete a 4,388 bp EST clone [GenBank: CK990464]. Primers were designed to the predicted translation start site on the genomic DNA in order to amplify fragments spanning the 5' end of the coding region from gut total cDNA. Sim4 [47] and Dotter [44] were used to align the cDNA sequence with the genomic DNA to identify exonic and intronic regions. The *zlg *cDNA was deposited under the GenBank accession number AY785950.

### Southern blot analysis

Liver genomic DNA from male Atlantic salmon and rainbow trout were isolated from 100 mg of tissue using the Easy-DNA Kit (Invitrogen). Southern blot analysis was performed as described by Hames and Higgins [48]. DNA was digested by restriction enzymes *Eco*R I, *Hind *III, *Bam*H I and *Bgl *II (NEB). The digested DNA was electrophoresed for 18 h and then transferred to Hybond, positively charged nylon membrane (Amersham).

Two probes were prepared to the 5' and 3' ends of the *zlg *cDNA sequence. Probe 1 included 206 nucleotides of the 3' end of the *zlg *ORF and 177 nucleotides of the 3'-UTR and probe 2 included 233 nucleotides of the VWD1 domain (Figure 1A). Both probes were gel purified and labeled with a Rediprime II random labeling kit (Amersham) with 50 μCi of α^32^P-labeled-dCTP.

Blots were prehybridized at 68°C for 4 h in hybridization buffer (5× SSC, 5× Denhardt's solution and 1% SDS) with 100 μg/mL denatured human placental DNA (Sigma). This was followed by replacement with fresh, preheated (68°C) hybridization buffer and the addition of the radiolabeled probe. Hybridization was allowed to proceed overnight. Following hybridization, the membrane was washed twice with 20 mL of 2× SSC, 0.1% SDS at room temperature for 15 min followed by two 15 min washes of 200 mL 0.2× SSC, 0.1% SDS at 65°C in a shaking bath. Prehybridization, hybridization and wash conditions were the same for both probes.

### Northern blot analysis

RNA was extracted from Atlantic salmon tissues (liver, brain, spleen, kidney, midgut, hindgut, foregut and gonads) and from zebrafish testes and gut using Trizol (Invitrogen). Total RNA samples were quantified and checked for quality by spectrophotometric analysis and agarose gel electrophoresis. Northern blots were prepared using the NorthernMax-Gly kit (Ambion) following the manufacturer's instructions. Ten μg of Atlantic salmon or 5 μg of zebrafish total RNA from each tissue was blotted on a Hybond positively charged nylon membrane (Amersham). Northern blot analysis of Atlantic salmon tissues utilized the same probe 1 described for the Southern blot analysis. The zebrafish zonadhesin probe was amplified from gut tissue using a primer set spanning from the 3' end of the epidermal growth factor domain through to the cytoplasmic domain using primers 5'-GGTTTGAGGGCACAAACTGT-3'and 5'-TAGGGATGCGCTGTCTTTTT-3'. Prehybridization for both Northern blots proceeded for 2 h at 42°C in 15 mL of ULTRAhyb buffer (Ambion). Hybridization with the α^32^P-dCTP-labeled probe at a final concentration of 10^6^cpm/mL of hybridization buffer was performed at 42°C overnight. The zebrafish Northern blot was stripped and reprobed with a probe designed from zebrafish alpha-tubulin that was expected to produce a doublet of 1485 bp [GenBank: AY398374] and 1544 bp [GenBank: AF029250].

### Semiquantitative reverse transcription PCR

Total RNA from Atlantic salmon and zebrafish tissue extracted as above was reverse transcribed using Superscript II enzyme (Invitrogen) and an 18 nucleotide oligo d(T) primer as described in the manufacturer's protocol with exception of the production of the cDNA template for the zebrafish MAM primer set which required a gene-specific internal primer for reverse transcription to reach this region (5'-AGACACTTTCACCCCCAGTG-3'). For Atlantic salmon, one μL of cDNA was amplified in a 25 μL reaction volume with either *zlg *probe 1 primers or ubiquitin primers (5'-ATGTCAAGGCCAAGATCCAG-3' and 5'-TAATGCCTCCACGAAGACG-3'). The *zlg *EST [GenBank: CK990464] and the 722P12 BAC were included as positive controls and both primer sets were run with template-free negative controls. For zebrafish, three primer sets were designed against the genomic *zan *sequence. The first primer set flanked the second MAM domain through to the first VWD domain (5'-TTGCAATTGATAGCGTCTGC-3' and 5'-TTCAGTCACAGGGTCACAGG-3'); the second primer set flanked the ninth VWD domain (5'-GGAGACCGTTACTGCAAACC-3' and 5'-CGAACAGTGATGCCGTACAC-3'); and the third primer set stretched from the epidermal growth factor domain to the cytoplasmic domain (see Northern blot probe description). The integrity of each cDNA was confirmed by control PCR reactions that used an ubiquitin primer set (5'-CCTCGAGGTAGAGCCAAGTG-3' and 5'-GCAGCACACAAGGTGCAAAGTA-3') and a template-free negative control.

### Puffer fish and zebrafish zonadhesin prediction and analysis

The puffer fish zonadhesin was found by BLASTN search of the three puffer fish genome assembles available at the MRC RFCGR Fugu genome database [33] with human and mouse *zan *nucleotide sequences. Scaffold 870 from assembly 2 was found to have similarity to zonadhesin and was subsequently analyzed by Genscan for coding sequences and peptide predictions. Puffer fish ESTs were aligned to scaffold 870 using Sim4.

The zebrafish zonadhesin was found by BLASTP search of the Ensembl zebrafish peptide database (Ensembl assembly 25.4.1) using a fragment of Atlantic salmon predicted protein as the query sequence. The Atlantic salmon query fragment consisted of all the amino acids except those representing the MAM domains. The two genomic regions identified were analyzed by Genscan to find the putative coding and protein sequences.

We looked for zonadhesin-like genes in puffer fish, zebrafish and chicken (*Gallus gallus*) genome projects using the ENSEMBL BLAST search tools using both cDNA and protein sequences from several zonadhesin-like genes as *in silico *probes. These included salmon *zlg*, human, puffer fish and zebrafish *zan*s and the related human *FCGBP *gene. Genomic regions from all significant matches were extracted and gene prediction analysis was performed using Genscan.

### Domain predictions and phylogenetic analysis

Protein domains were predicted using SMART and InterPro prediction tools and the domains were extracted from the parent nucleotide and protein sequences. Multiple sequence alignments of the extracted domains were done using ClustalX [50] followed by manual inspection. See additional file 1 and additional file 2 for VWD and MAM multiple alignments respectively. Neighbor-joining trees were created using MEGA3. Consensus trees based on 1000 pseudoreplicates are reported with the bootstrap support values indicated above the respective nodes. Gaps were removed and we reported phylogenetic data using the Poisson correction model with uniform rates across all sites. Neighbor-joining trees were also performed using the Poisson correction model with unequal rates across sites using gamma distance parameters 0.65 and 2.25. While some of the less supported nodes changed, the clades discussed here did not vary substantially using these different parameters for either the MAM or the VWD trees. We also used the equal input model using either uniform rates across all sites or unequal rates across all sites using gamma distance parameters 0.65 and 2.25. Again these parameters did not change the topology of the clades discussed in the text.

## Authors' contributions

PNDH carried out the molecular genetic studies, sequencing and sequence alignment and drafted the manuscript. MDW performed the comparative genomic analysis and contributed to the experimental design and manuscript draft. KRVS conceived the study and participated in experimental genetic studies. WSD contributed to the production of the GRASP EST database. BFK participated in study design and coordination. All authors read and approved the final manuscript.

## Supplementary Material

Additional File 1 Multiple sequence alignment (Fasta format) of VWD domains used to construct the phylogeny in Figure 6. Click here for file

Additional File 2 Multiple sequence alignment (Fasta format) of MAM domains used to construct the phylogeny in Figure 6. Click here for file
